# Advances in Electromembrane Processes for Resource Recovery

**DOI:** 10.3390/membranes16030111

**Published:** 2026-03-20

**Authors:** Krzysztof Mitko, Marian Turek, Mònica Reig, Xanel Vecino

**Affiliations:** 1 Faculty of Chemistry, Silesian University of Technology, ul. B. Krzywoustego 6, 44-100 Gliwice, Poland; marian.turek@polsl.pl; 2 Chemical Engineering Department, Escola d’Enginyeria de Barcelona Est (EEBE), Universitat Politècnica de Catalunya (UPC)-BarcelonaTECH, Av. Eduard Maristany 16, Campus Diagonal-Besòs, 08019 Barcelona, Spain; monica.reig@upc.edu; 3 Barcelona Research Center for Multiscale Science and Engineering (CCEM), Av. Eduard Maristany 16, Campus Diagonal-Besòs, 08019 Barcelona, Spain; 4 Chemical Engineering Department, School of Industrial Engineering (EEI), University of Vigo, 36310 Vigo, Spain; xanel.vecino@uvigo.gal

Electromembrane processes are a separate class of membrane methods that utilize ion transport across the ion exchange membranes. Electromembrane processes have been applied to simultaneously demineralize and concentrate solutions of ionic species since the 1940s [1]. However, the improvements in membrane permselectivity and conductivity made in the 1950s [2,3] actually launched the industrial applications of electromembrane processes. Since then, the electromembrane processes have found numerous applications, including brackish water desalination [4], edible salt production from seawater [5], chemical production [6], heavy metal removal [7], desalination of process streams in the food industry [8], and, finally, resource recovery.

Resource recovery is a necessary step to decrease dependence on non-renewable raw materials and minimize the water stress caused by population growth and industrial development. The electromembrane process, which is able to selectively separate ionic species from non-ionic species, plays a role in developing resource recovery technologies. The Special Issue of *Membranes*, entitled “Advances in Electromembrane Processes for Resource Recovery”, presents recent research in this field.

All electromembrane processes rely on the migration of ions; thus, understating of this phenomenon is crucial in designing electromembrane-based technologies for resource recovery. However, the mathematical models used to describe these processes become increasingly difficult to solve numerically under overlimiting conditions at high concentrations. For this reason, Kovalenko et al. (contribution 1) proposed a new mathematical model that describes the diluate channel of an electromembrane stack. The new model, a hybrid of analytical and numerical solutions, can be used to solve other problems of membrane electrochemistry, where a quasi-equilibrium layer makes it difficult to use only numerical methods. The research offers a simple formula for engineering calculations of the acceptable potential at a given concentration.

The most common electromembrane process is electrodialysis (ED). Miśkiewicz et al. (contribution 2) investigated the application of electrodialysis in processing the liquid low-level radioactive waste. The results show that electrodialysis is suitable not only for desalination but also for decontaminating the liquid waste, as it is very effective in removing ^137^Cs, ^60^Co, ^85^Sr, and ^241^Am, isotopes responsible for radioactivity. Simultaneously, electrodialysis did not affect the concentration of octylphenyl ethoxylate (Triton X-102), allowing the selective recovery of this organic species. This study shows that electromembrane processes can facilitate the recovery of resources (water, organic species) from hazardous waste streams, including radioactive waste.

Many branches of industry are reliant on platinum group metals (PGMs), and as the demand for these scarce resources grows, their recovery is being widely investigated. Electromembrane methods can be utilized in PGM recovery, as showcased by Zimmermann et al. (contribution 3), who investigated the recovery of palladium using electrodialysis. In their proof-of-concept study, they compared two ion exchange membranes, Selemion AMVN/CMVN and Fujifilm Type 12 AEM/CEM. The researchers identified potential challenges in applying electrodialysis for palladium recovery from acidic solutions: low current efficiency, water transport, and membrane stability.

Ion exchange membranes can be modified to increase the selectivity towards the univalent ions. Such membranes were utilized by Haddad et al. (contribution 4) to investigate the treatment of ion exchange post-regeneration lyes using a standalone selective ED and an integrated selective ED-direct contact membrane distillation system. The idea was to recover NaCl for reuse in ion exchange regeneration, as well as recover fresh water and natural organic matter for agricultural purposes. The study confirmed that 55–85% of ion-exchange resin capacity can be restored when using recovered NaCl, showcasing how the electromembrane processes can increase the circularity of water treatment systems. This study also demonstrates how an electromembrane process can be integrated with other membrane processes to increase product recovery.

The recovery of organic species is an important application of conventional electrodialysis. Volatile fatty acids (VFAs) are of significant importance, as they are widely used in the industry. VFAs are mostly produced from fossil fuels, and while bioproduction can be an alternative route, it requires better separation of a post-reaction mixture. For this reason, Caveriviere et al. (contribution 5) studied the selectivity of acetate and butyrate recovery using chloride as the reference anion. The researchers also tested membranes selective towards univalent anions. They concluded that in the range of conditions investigated, permselectivity depended only on the membrane and solute, but not on the concentration or proportions of anions in the feed.

While ED is the most common electromembrane process, others can be utilized for resource recovery. Koomson et al. (contribution 6) studied a coupled microbial desalination cell–microbial electrolysis cell (MDC-MEC) process. The proposed system treats water bodies polluted by d-block metal ions and ammonia: Pb^2+^ and Fe^2+^ are removed by reduction on the MEC cathode and by biosorption in the MDC, while the nitrogen (as NH_4_^+^) is removed by the MDC. Such a bio-electrochemical coupled system decreases the energy consumption by using the microbial cell as a power source. The study showcases the versatility of ion exchange membranes, as they can be applied to both classical electrochemical and bio-electrochemical systems.

Electromembrane processes can also be hybridized with pressure-driven processes. Butylskii et al. (contribution 7) presented a hybrid stack made of heterogeneous ion exchange membranes and a porous membrane, either a polymeric or ceramic one, with a pressure difference maintained between two sides of the porous membrane. Such a hybrid electrobaromembrane system has been proven to be very efficient in separating chloride and dihydrogen phosphate anions.

Electric energy is one of the resources that can be used in electromembrane processes. Reverse electrodialysis (RED) can harness the Gibbs free energy of mixing two solutions of different salinity. However, RED requires pretreatment if natural water is utilized as feed. For this reason, Ju et al. (contribution 8) investigated several RED pretreatment options using real brackish water reverse osmosis (BWRO) brine from a wastewater reclamation plant: cartridge filters; micro-, ultra-, and nanofiltration; activated filter media; and granular activated carbon. The researchers concluded that while nanofiltration is the most efficient pretreatment, its high energy consumption cuts into the net power generated by the RED. This study showcases that during the design of the electromembrane process, it is important to not only focus on the electromembrane unit alone but also take a holistic view of the entire plant, including the pretreatment.

In summary, this Special Issue offers insight into various aspects of the application of electromembrane processes in resource recovery: from mathematical modelling to practical applications; from conventional electrodialysis to novel hybrid processes; from focusing on the electromembrane stack to investigating the effect of pretreatment. The broad range of issues discussed in this Special Issue reflects the variety of applications of electromembrane processes.
